# Efficacy and Safety of Apixaban, Dabigatran, Rivaroxaban, and Warfarin in Asians With Nonvalvular Atrial Fibrillation

**DOI:** 10.1161/JAHA.117.008150

**Published:** 2018-04-05

**Authors:** Yi‐Hsin Chan, Lai‐Chu See, Hui‐Tzu Tu, Yung‐Hsin Yeh, Shang‐Hung Chang, Lung‐Sheng Wu, Hsin‐Fu Lee, Chun‐Li Wang, Chang‐Fu Kuo, Chi‐Tai Kuo

**Affiliations:** ^1^ Cardiovascular Department Chang Gung Memorial Hospital Linkou, Taoyuan Taiwan; ^2^ Division of Rheumatology, Allergy and Immunology Department of Internal Medicine Chang Gung Memorial Hospital Linkou, Taoyuan Taiwan; ^3^ College of Medicine Chang Gung University Taoyuan Taiwan; ^4^ Department of Public Health College of Medicine Chang Gung University Taoyuan Taiwan; ^5^ Biostatistics Core Laboratory Molecular Medicine Research Center Chang Gung University Taoyuan Taiwan

**Keywords:** atrial fibrillation, direct thrombin inhibitor, factor Xa inhibitor, hemorrhage, ischemic stroke, mortality, warfarin, Atrial Fibrillation, Ischemic Stroke, Intracranial Hemorrhage

## Abstract

**Background:**

Whether non–vitamin K antagonist oral anticoagulants (NOACs) are superior to warfarin among Asians with nonvalvular atrial fibrillation remains unclear.

**Methods and Results:**

In this nationwide retrospective cohort study collected from Taiwan National Health Insurance Research Database, there were 5843, 20 079, 27 777, and 19 375 nonvalvular atrial fibrillation patients taking apixaban, dabigatran, rivaroxaban and warfarin, respectively, from June 1, 2012 to December 31, 2016. Propensity‐score weighting was used to balance covariates across study groups. Patients were followed until the first occurrence of any efficacy or safety outcome or the end date of study. Hazard ratios (95% confidence intervals) comparing apixaban, dabigatran, and rivaroxaban with warfarin were: ischemic stroke/systemic embolism (IS/SE), 0.55 (0.43–0.69), 0.82 (0.68–0.98), and 0.81 (0.67–0.97); major bleeding, 0.41 (0.31–0.53), 0.65 (0.53–0.80), and 0.58 (0.46–0.72); and all‐cause mortality, 0.58 (0.51–0.66), 0.61 (0.54–0.68), and 0.57 (0.51–0.65). A total of 3623 (62%), 17 760 (88%), and 26 000 (94%) patients were taking low‐dose apixaban (2.5 mg twice daily), dabigatran (110 mg twice daily), and rivaroxaban (10–15 mg once daily), respectively. Similar to all‐dose NOACs, all low‐dose NOACs had lower risk of IS/SE, major bleeding, and mortality when compared with warfarin. In contrast to other standard‐dose NOACs, apixaban was associated with lower risks of IS/SE (0.45 [0.31–0.65]), major bleeding (0.29 [0.18–0.46]), and mortality (0.23 [0.17–0.31]) than warfarin.

**Conclusions:**

All NOACs were associated with lower risk of IS/SE, major bleeding, and mortality compared with warfarin in the largest real‐world practice among Asians with nonvalvular atrial fibrillation. All low‐dose NOACs had lower risk of IS/SE, major bleeding, and mortality when compared with warfarin. Standard‐dose apixaban caused a lower risk of IS/SE, major bleeding, and mortality compared with warfarin.

**Clinical Perspective**

**What Is New?**

- This is the largest population‐based study of Asians with nonvalvular atrial fibrillation to investigate the effectiveness and safety of the non–vitamin K antagonist oral anticoagulants apixaban, dabigatran, and rivaroxaban versus warfarin during an extended follow‐up period.
- Our results showed that all three non–vitamin K antagonist oral anticoagulants exhibited lower risks of thromboembolic events, all major bleeding, and all‐cause mortality compared with warfarin.
- There is a high prevalence of low‐dose non–vitamin K antagonist oral anticoagulant use among this large Asian cohort, and this subgroup also had lower risks of thromboembolic events, major bleeding, and mortality compared to warfarin.

**What Are the Clinical Implications?**

- Apixaban, rivaroxaban, and dabigatran should be considered as first line therapy for Asian patients with nonvalvular atrial fibrillation.

## Introduction

Atrial fibrillation (AF) is the most common cardiac arrhythmia with a global prevalence of 2% to 3%. AF significantly increases the risk of thromboembolic events and death.^1^ Oral anticoagulants like vitamin K antagonists (eg, warfarin) or non–vitamin K antagonist oral anticoagulants (NOACs; eg, dabigatran, rivaroxaban, apixaban, and edoxaban) are indicated for stroke/systemic embolism prevention in AF patients with 1 or more risk factors for stroke. Several large trials have suggested that NOACs have similar or improved efficacy compared with warfarin and are more convenient and safer alternatives to warfarin.^2^, ^3^, ^4^, ^5^ The safety profiles showed that all NOACs caused a lower risk of intracranial hemorrhage, but an increased risk of gastrointestinal bleeding with rivaroxaban, edoxaban, and dabigatran (150 mg twice daily) compared with warfarin. Of particular note, Asians may receive greater benefit from NOACs compared with non‐Asians, as they carry a higher risk of intracranial hemorrhage and have a greater difficulty maintaining the therapeutic range of international normalized ratio of 2 to 3 when taking warfarin.^6^, ^7^ The subgroup analyses from 4 pivotal NOAC trials indicated that NOACs may be more effective and safer in Asians than in non‐Asians.^8^, ^9^ Also, a recent real‐world study showed that dabigatran and rivaroxaban have favorable efficacy and safety profiles compared with warfarin in a large nationwide Asian cohort with nonvalvular AF (NVAF).^10^ However, the follow‐up periods and patient numbers in those real‐world studies were limited, and the efficacy and safety profiles of apixaban were lacking. As we know, there has been only 1 publication that directly compares the efficacy and safety of all 3 NOACs (ie, apixaban, dabigatran, and rivaroxaban) in a limited number of Asians with AF.^11^ The objective of this study was to compare the efficacy and safety of apixaban, dabigatran, and rivaroxaban with warfarin in Asians with NVAF as a nationwide retrospective cohort study with an extended follow‐up period.

## Methods

The data, analytical methods, and study materials will not be made available to other researchers for purposes of reproducing the results or replicating the procedure.

### Study Population

This study was approved by the Institutional Review Board of Chang Gung Memorial Hospital. Informed consent was waived by the Institutional Review Board of Chang Gung Memorial Hospital because the original identification number of each patient in the National Health Insurance Research Database (NHIRD) was encrypted and deidentified to protect patients' privacy by using a consistent encrypting procedure. The National Health Insurance system is a mandatory universal health insurance program in Taiwan that provides comprehensive medical care coverage to all Taiwanese. As of 2014, there were >23 million enrollees and a >99% coverage rate of the entire population.^12^

### Study Design

**Figure 1:**
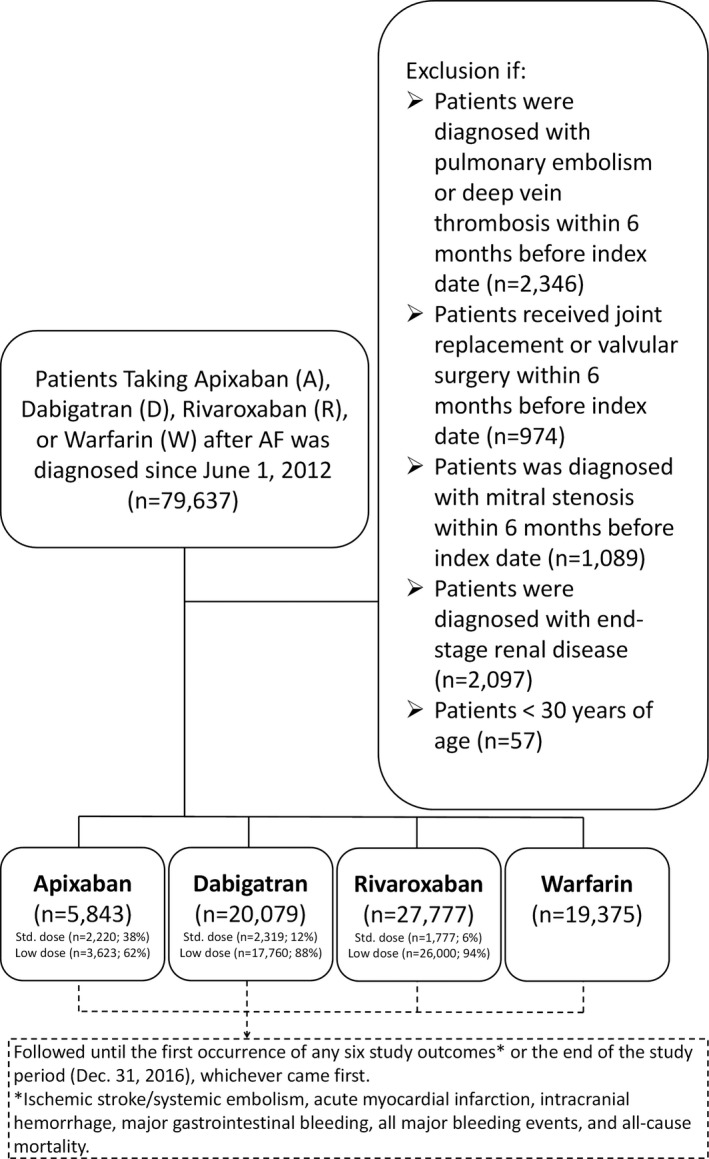
Enrollment of patients with non‐valvular AF. From June 1, 2012, to December 31, 2016, 5843, 20 079, and 27 777 nonvalvular AF patients receiving the apixaban, dabigatran, and rivaroxaban and 19 375 patients prescribed warfarin were enrolled in this study. AF indicates atrial fibrillation.

A dynamic cohort with 4 study groups (apixaban, dabigatran, rivaroxaban, and warfarin) was used in the study. A flowchart of the study enrollment is shown in Figure 1. A total of 279 776 patients diagnosed with AF (*International Classification of Diseases, Ninth Revision, Clinical Modification [ICD‐9‐CM]* codes [427.31] from January 1, 2010, to December 31, 2015, or *ICD‐10‐CM* codes [I48] from January 1, 2016, to December 31, 2016) were identified. Patients were included who had their first prescription of an NOAC including dabigatran (approval date: June 1, 2012), rivaroxaban (approval date: February 1, 2013), or apixaban (approval date: June 1, 2014), as well as patients who started warfarin treatment from June 1, 2012, to December 31, 2016. The index date was defined as the first date of prescription for any NOAC or warfarin after June 1, 2012, for each group. The follow‐up period was defined from the index date until the first occurrence of any study outcome or the end date of study period (December 31, 2016), whichever came first.

### Exclusion Criteria

Patients were excluded who took more than 1 kind of NOAC during their entire treatment course. To establish a cohort of NVAF patients who took oral anticoagulants for the primary purpose of stroke prevention, those patients were excluded with diagnoses indicating valvular AF (mitral stenosis or valvular surgery), venous thromboembolism (pulmonary embolism or deep vein thrombosis) or joint replacement therapy within 6 months before the index date. Patients were also excluded who had end‐stage renal disease requiring renal replacement therapy because NOACs are contraindicated in such patients in Taiwan. The diagnosis of end‐stage renal disease requiring dialysis in our present study was confirmed by both specific *ICD‐9‐CM* or *ICD‐10‐CM* codes and enrollment in the Registry of Catastrophic Illness Patient Database, a subpart of the National Health Insurance database. Only patients with end‐stage renal disease on dialysis can be registered in the Registry of Catastrophic Illness Patient Database according to the rules of the National Health Insurance system.^13^, ^14^

### Study Outcomes

Six study outcomes were defined to determine the efficacy and safety profiles for NOACs and warfarin: ischemic stroke/systemic embolism (IS/SE), intracranial hemorrhage (ICH), major gastrointestinal bleeding (GIB), acute myocardial infarction, all major bleeding events, and all‐cause mortality. All study outcomes were required to be a discharge diagnosis to avoid misclassification. The accuracy of diagnosis indicating IS in the NHIRD was validated previously.^15^, ^16^ In addition, we also validated the *ICD‐9‐CM* codes for identifying IS by analyzing the medical records of 1002 consequent AF patients in the inpatient claims database of Chang Gung Memorial Hospital, Linkou, which is the largest medical center in Taiwan, between January 2010 and December 2015. Clinical diagnosis of IS was determined according to the compatible brain imaging. In total, there were 104 confirmed cases of IS at discharge, and 96 can be identified with an *ICD‐9‐CM* code indicating IS (433, 434, or 436) in the discharge claims. There were only 10 cases with *ICD‐9‐CM* indicating IS without compatible brain imaging available. The sensitivity, specificity, positive predictive value, and negative predictive value were 0.92, 0.98, 0.91 and 0.99, respectively. ICH was defined with the use of codes for atraumatic hemorrhage. Major GIB was defined as a hospitalized primary code indicating bleeding in the gastrointestinal tract. All major bleeding events were defined as the total hospitalized events of ICH, major GIB, and other critical site bleedings. The diagnosis codes of NHIRD were shifted from *ICD‐9‐CM* to *ICD‐10‐CM* after January 1, 2016. The *ICD‐9‐CM* and *ICD‐10‐CM* codes used to identify the study outcomes and the baseline covariates are summarized in Table 1. The same patient may have had more than 1 study outcome during the study duration, but only the study outcome that first occurred was considered in the study. For those patients with a thromboembolic event or major bleeding later in the hospitalization leading to death, both the thromboembolic event/major bleeding and mortality were reported individually.

**Table 1:** *ICD‐9‐CM* and *ICD‐10‐CM* Codes Used to Define the Comorbidities and Clinical Outcome in the Study Cohort

Disease	*ICD‐9* Codes	*ICD‐10* Codes	Diagnosis Definition
Atrial fibrillation	427.31	I48	Discharge or outpatient department ≥2
Ischemic stroke	433, 434, 436	I63, I64	Discharge
Transient ischemic attack	435	G45	Discharge
Peripheral arterial occlusive disease	440.2	I70.2–I70.9, I71; I73.9	Discharge
Myocardial infarction	410, 411, 412	I21–I25	Discharge
Congestive heart failure	428	I11.0, I13.0, I13.2, I42.0, I50, I50.1, I50.9	Discharge
Hypertension	401, 402	I10–I16	Outpatient department ≥2
Diabetes mellitus	250	E10.0, E10.1, E10.9, E11.0, E11.1, E11.9	Outpatient department ≥2
Hyperlipidemia	272	E78	Outpatient department ≥2
Chronic gout	274.0, 274.10, 274.11, 274.19, 274.81, 274.82, 274.89, 274.9	M10, M1A	Outpatient department ≥2
Chronic lung disease	490, 491.0, 491.1, 491.20–491.22, 491.8, 491.9, 492.0, 492.8, 493.00–493.02 493.10–493.12, 493.20–493.22, 493.81, 493.82, 493.90–493.92, 494.0, 494.1, 495.8, 495.9, 496, 500, 502, 503, 504, 505, A323, A325	J44	Discharge
Chronic kidney disease	580–589	I12, I13, N00, N01, N02, N03, N04, N05, N07, N11, N14, N17, N18, N19, Q61	Outpatient department ≥2
Chronic liver disease	570, 571, 572	B150, B160, B162, B190, K704, K72, K766, I85	Outpatient department ≥2
Malignancy	140.0–208.9	C	Outpatient department ≥2
Intracranial hemorrhage	430, 431, 432, 852, 853	I60, I61, I62	Discharge
Gastrointestinal bleeding	456.0, 456.2, 455.2, 455.5, 455.8, 530.7, 530.82, 531.0–531.6, 532.0–532.6, 533.0–533.6, 534.0–534.6, 535.0–535.6 537.83, 562.02, 562.03, 562.12 562.13 568.81, 569.3, 569.85, 578.0, 578.1, 578.9	K250, K260, K270, K280, K290	Discharge
Other critical site bleeding	423,0, 459.0, 568.81, 593.81, 599.7, 623.8, 626.32, 626.6, 719.1, 784.7, 784.8, 786.3	D62, J942, H113, H356, H431, N02, N95, R04, R31, R58	Discharge

*ICD‐9‐CM* indicates *International Classification of Disease, 9th Edition, Clinical Modification; ICD‐10‐CM, International Classification of Disease, 10th Edition, Clinical Modification*.

### Covariates

Baseline covariates were referred to any claim record with the above diagnoses or medication codes before the index date. A bleeding history was confined to events within 6 months preceding the index date. A history of prescription for medicine was confined to at least once within 3 months preceding the index date.^17^ The CHA_2_DS_2_‐VASc score (congestive heart failure, hypertension, age 75 years or older, diabetes mellitus, previous stroke or transient ischemic attack, vascular disease, age 65–74 years, female sex) was adopted to predict the risk of ischemic stroke/thromboembolic events in AF patients, and the HAS‐BLED score (hypertension, abnormal renal or liver function, stroke, bleeding history, labile international normalized ratio, age 65 years or older, and antiplatelet drug or alcohol use) was adopted to predict the risk of bleeding in patients with AF treated with oral anticoagulants.^18^, ^19^

### Statistical Analysis

The propensity score method, which simulates the effect of a randomized clinical trial for observational cohort data,^20^ was used to estimate the 6 study outcomes of 3 NOACs and warfarin. Inverse probability of treatment weights of propensity scores was used to balance covariates across the 4 groups. Generalized boosted models were used, based on 5000 regression trees, to calculate weights for optimal balance among 4 study groups.^21^ The weights were derived to obtain estimates representing average treatment effects in the treated patients. The covariates in Table 2 were included in the propensity models, except for CHA_2_DS_2_‐VASc and HAS‐BLED scores, because CHA_2_DS_2_‐VASc and HAS‐BLED scores were a combination of other covariates. Incidence rates were estimated using the total number of study outcomes during the follow‐up period divided by person‐years at risk. The risk of study outcomes for 3 NOACs versus warfarin (reference) was obtained using survival analysis (Kaplan‐Meier method and log‐rank test for univariate analysis and time‐dependent Cox proportional hazards regression for multivariate analysis). The balance of potential confounders at baseline (index date) between each study group was assessed using the absolute standardized mean difference rather than statistical testing, because balance is a property of the sample and not of an underlying population. The value of absolute standardized mean difference ≤0.1 indicated an insignificant difference in potential confounders between the 2 study groups.^20^ Statistical significance was defined as a *P* value <0.05. All statistical analyses were performed using computer software (SAS 9.4; SAS Institute Inc, Cary, NC).

**Table 2:** Baseline Characteristics of Patients With NVAF Taking Oral Anticoagulants Before and After Propensity Score Weighting

	Patient Baseline Characteristics							
	Before Propensity Score Weighting				After Propensity Score Weighting			
	Apixaban (n=5843)	Dabigatran (n=20 079)	Rivaroxaban (n=27 777)	Warfarin (n=19 375)	Apixaban	Dabigatran	Rivaroxaban	Warfarin
Age, y	76±10	75±10	75±10	71±13	76±10	76±10	76±10	76±10
Female	45% (2629)	40% (8018)	45% (12 403)	42% (8154)	45%	45%	45%	46%
CHA_2_DS_2_‐VASc	3.89±1.56	3.74±1.52	3.83±1.57	3.26±1.81	3.89±1.56	3.88±0.82	3.89±0.71	3.89±0.88
HAS‐BLED	2.96±1.12	2.83±1.08	2.91±1.10	2.64±1.29	2.96±1.12	2.96±0.59	2.96±0.51	2.97±0.61
Chronic lung disease	13% (780)	12% (2323)	14% (3816)	13% (2494)	13%	13%	14%	14%
Chronic liver disease	16% (929)	14% (2831)	16% (4421)	16% (3048)	16%	16%	16%	16%
Chronic kidney disease	29% (1671)	20% (3922)	24% (6786)	24% (4702)	29%	28%	28%	29%
Congestive heart failure	13% (735)	11% (2172)	13% (3582)	14% (2699)	13%	12%	13%	13%
Hypertension	87% (5055)	84% (16 863)	86% (23 766)	78% (15 099)	87%	87%	86%	87%
Hyperlipidemia	54% (3161)	50% (10 033)	53% (14 747)	45% (8742)	54%	54%	54%	54%
Diabetes mellitus	41% (2389)	38% (7647)	39% (10 752)	36% (6948)	41%	41%	41%	40%
Previous stroke	20% (1173)	24% (4778)	20% (5675)	15% (2936)	20%	20%	20%	20%
Previous TIA	3% (167)	3% (573)	2% (667)	2% (344)	3%	3%	3%	3%
Ischemic heart disease	13% (733)	10% (1961)	12% (3399)	11% (2098)	13%	13%	13%	12%
Gout	25% (1453)	23% (4525)	24% (6779)	23% (4496)	25%	25%	25%	25%
Peripheral artery disease	0% (4)	0% (11)	0% (19)	0% (16)	0%	0%	0%	0%
Malignancy	10% (555)	8% (1687)	9% (2518)	8% (1581)	10%	9%	9%	10%
History of bleeding	2% (113)	2% (415)	2% (644)	2% (451)	2%	2%	2%	2%
Use of NSAIDs	27% (1556)	22% (4401)	24% (6657)	25% (4792)	27%	27%	27%	26%
Use of PPI	11% (655)	8% (1654)	11% (2906)	13% (2421)	11%	11%	11%	11%
Use of ACEI/ARB	6% (329)	28% (5631)	19% (5179)	28% (5383)	6%	6%	6%	6%
Use of H_2_ blocker	31% (1810)	29% (5772)	29% (8175)	32% (6200)	31%	31%	31%	32%
Use of amiodarone	28% (1649)	22% (4498)	27% (7370)	39% (7472)	28%	28%	28%	28%
Use of dronedarone	5% (286)	2% (372)	5% (1281)	2% (464)	5%	5%	5%	5%
Use of β‐blocker	59% (3451)	54% (10 839)	57% (15 782)	61% (11 824)	59%	59%	59%	59%
Use of diltiazem/verapamil	25% (1432)	23% (4565)	24% (6779)	27% (5293)	25%	24%	24%	24%
Use of digoxin	20% (1149)	24% (4832)	23% (6248)	30% (5882)	20%	20%	20%	20%
Use of statin	4% (229)	20% (4101)	14% (3949)	17% (3322)	4%	4%	4%	4%
PCI	7% (415)	5% (916)	6% (1750)	5% (1051)	7%	7%	7%	7%
CABG	1% (31)	0% (40)	0% (104)	1% (143)	1%	0%	0%	1%

ACEI indicates angiotensin‐converting enzyme inhibitor; ARB, angiotensin II receptor antagonists; CABG, coronary artery bypass graft; CHA_2_DS_2_‐VASc, congestive heart failure, hypertension, age 75 years or older, diabetes mellitus, previous stroke/transient ischemic attack, vascular disease, age 65 to 74 years, female; HAS‐BLED, hypertension, abnormal renal or liver function, stroke, bleeding history, labile INR, age 65 years or older, and antiplatelet drug or alcohol use (labile INR could not be determined from claims and was excluded from our scoring); NSAIDs, nonsteroidal anti‐inflammatory drugs; NVAF; nonvalvular atrial fibrillation; PCI, percutaneous coronary intervention; PPI, proton pump inhibitor; and TIA, transient ischemic attack.

## Results

A total of 5843, 20 079, 27 777, and 19 375 consecutive patients taking apixaban, dabigatran, rivaroxaban, and warfarin, respectively, from June 1, 2012, to December 31, 2016, were enrolled. The mean follow‐up periods were 0.76, 1.55, 1.24, and 1.47 years for apixaban, dabigatran, rivaroxaban, and warfarin, respectively. Apixaban had the shortest mean follow‐up period owing to its introduction later to the market. In general, all NOAC patient groups were older, had higher CHA_2_DS_2_‐VASc and HAS‐BLED scores, and had a higher proportion of comorbidities than the warfarin group before propensity score weighting (Table 2). After propensity score weighting, the 4 study groups were well balanced in most characteristics (absolute standardized mean difference <0.1, data not shown) (Table 2).

**Figure 2:**
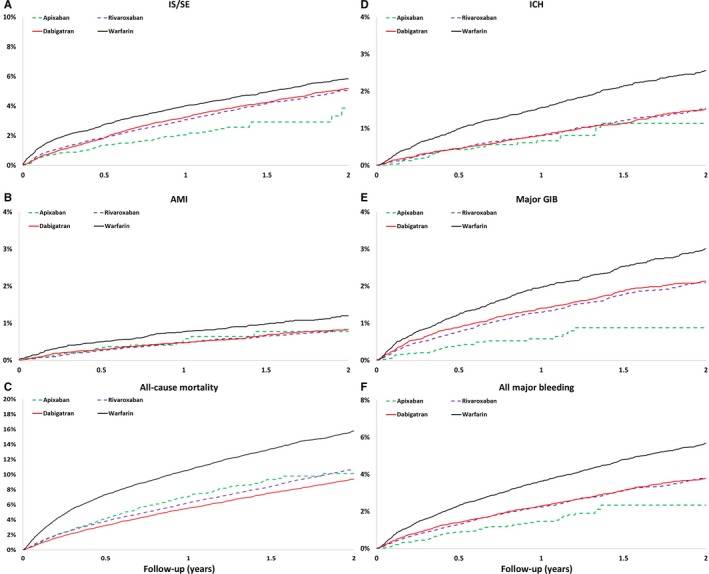
The cumulative incidence curves of IS/SE (A), AMI (B), all‐cause mortality (C), ICH (D), major GIB (E), and all major bleeding (F) for patients with nonvalvular AF taking oral anticoagulants before propensity score weighting. Apixaban, rivaroxaban, and dabigatran are associated with reduced risk of IS/SE, ICH, major GIB, all major bleeding, and all cause‐mortality compared with warfarin. AF indicates atrial fibrillation; AMI, acute myocardial infarction; GIB, gastrointestinal bleeding; ICH, intracranial hemorrhage; IS/SE, ischemic stroke/systemic embolism.

**Figure 3:**
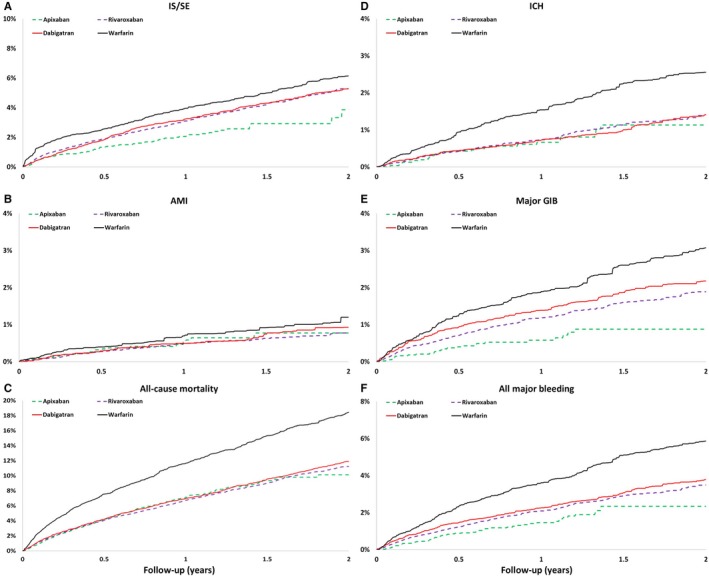
The cumulative incidence curves of IS/SE (A), AMI (B), all‐cause mortality (C), ICH (D), major GIB (E), and all major bleeding (F) for patients with nonvalvular AF taking oral anticoagulants after propensity score weighting. Apixaban, rivaroxaban, and dabigatran are associated with reduced risk of IS/SE, ICH, all major bleeding, and all cause‐mortality compared with warfarin. AF indicates atrial fibrillation; AMI, acute myocardial infarction; GIB, gastrointestinal bleeding; ICH, intracranial hemorrhage; IS/SE, ischemic stroke/systemic embolism.

**Figure 4:**
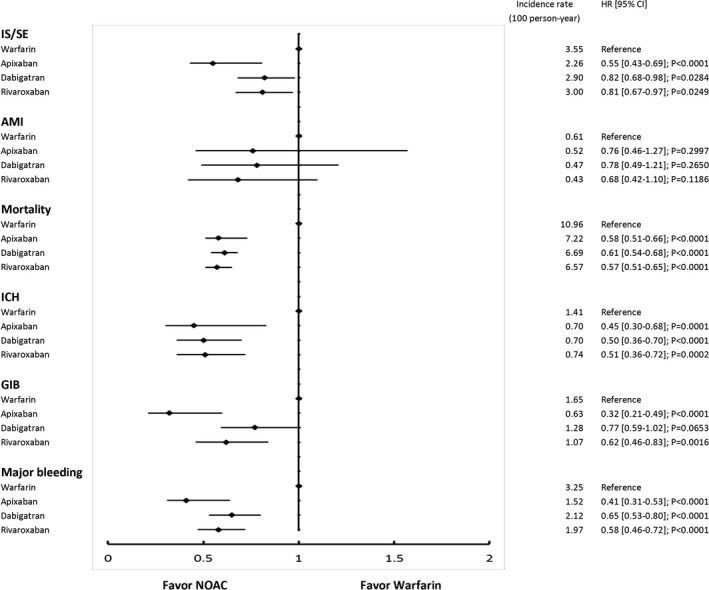
The forest plot of hazard ratios for each NOAC vs warfarin comparison. Apixaban, rivaroxaban, and dabigatran are associated with reduced risks of IS/SE, ICH, all major bleeding, and all cause‐mortality compared with warfarin. AMI indicates acute myocardial infarction; CI, confidence interval; GIB, gastrointestinal bleeding; HR, hazard ratio; ICH, intracranial hemorrhage; IS/SE, ischemic stroke/systemic embolism; NOAC, non–vitamin K antagonist oral anticoagulant.

The cumulative risk showed clear separation of event curves for IS/SE, ICH, GIB, all major bleeding, and all‐cause mortality for all NOACs versus warfarin either before or after adjustment (Figures 2 and 3). All NOACs had a lower risk of IS/SE, ICH, major GIB, all major bleeding, and all‐cause mortality compared with warfarin (Figure 4). The lowest incidence rate of IS/SE occurred with apixaban (2.26%/year), followed by the rate of dabigatran (2.90%/year) and rivaroxaban (3.00%/year), and the highest occurred with warfarin (3.55%/year) (hazard ratios, HRs [95% confidential interval, CI], 0.55 [0.43–0.69] for apixaban versus warfarin; 0.82 [0.68–0.98] for dabigatran versus warfarin; 0.81 [0.67–0.97] for rivaroxaban versus warfarin). Apixaban (0.70%/year), dabigatran (0.70%/year), and rivaroxaban (0.74%/year) were all associated with lower incidence rates of ICH compared with warfarin (1.41%/year) (HRs [95% CI], 0.45 [0.30–0.68] for apixaban versus warfarin; 0.50 [0.36–0.70] for dabigatran versus warfarin; 0.51 [0.36–0.72] for rivaroxaban versus warfarin). Apixaban (1.52%/year), dabigatran (2.12%/year), and rivaroxaban (1.97%/year) were all associated with lower incidence rates of major bleeding compared with warfarin (3.25%/year) (HRs [95% CI], 0.41 [0.31–0.53] for apixaban versus warfarin; 0.65 [0.53–0.80] for dabigatran versus warfarin; 0.58 [0.46–0.72] for rivaroxaban versus warfarin). Apixaban (7.22%/year), dabigatran (6.69%/year), and rivaroxaban (6.57%/year) all have lower incidence rates of all‐cause mortality than warfarin (10.96%/year) (HRs [95% CI], 0.58 [0.51–0.66] for apixaban versus warfarin; 0.61 [0.54–0.68] for dabigatran versus warfarin; 0.57 [0.51–0.65] for rivaroxaban versus warfarin) (Figure 4 and Table 3).

**Table 3:** Number of Events, Crude and Adjusted Event Rates Among 4 Treatment Groups

	Apixaban (n=5843)			Dabigatran (n=20 079)			Rivaroxaban (n=27 777)			Warfarin (n=19 375)		
	Events	Crude Incidence^a^	Adjusted Incidence^b^	Events	Crude Incidence^a^	Adjusted Incidence^b^	Events	Crude Incidence^a^	Adjusted Incidence^b^	Events	Crude Incidence^a^	Adjusted Incidence^b^
Ischemic stroke/systemic embolism	100	2.26	2.26	854	2.74	2.90	977	2.84	3.00	929	3.26	3.55
Ischemic stroke	91	2.06	2.06	747	2.40	2.56	870	2.53	2.65	793	2.79	3.05
Acute myocardial infarction	23	0.52	0.52	134	0.43	0.47	146	0.42	0.43	177	0.62	0.61
All‐cause mortality	319	7.22	7.22	1575	5.05	6.69	2051	5.97	6.57	2588	9.09	10.96
Intracranial hemorrhage	31	0.70	0.70	233	0.75	0.70	272	0.79	0.74	378	1.33	1.41
Major gastrointestinal bleeding	28	0.63	0.63	362	1.16	1.28	394	1.15	1.07	444	1.56	1.65
All major bleeding	67	1.52	1.52	625	2.01	2.12	707	2.06	1.97	855	3.00	3.25

a Events divided by 100 person‐years.

b Inverse probability of treatment weighted to apixaban and expressed as population average treatment rates per 100 years.

**Figure 5:**
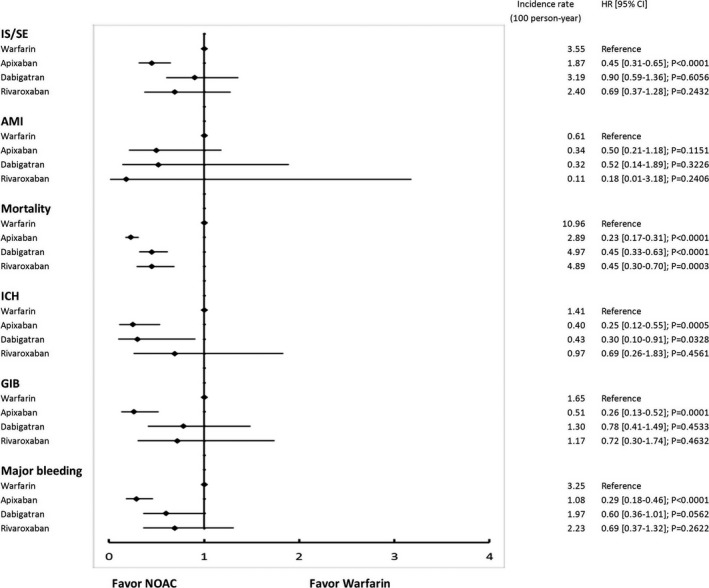
The forest plot of hazard ratios for each standard‐dose NOAC (apixaban 5 mg twice daily, dabigatran 150 mg twice daily, and rivaroxaban 20 mg once daily) vs warfarin comparison. Standard‐dose apixaban is associated with lower risks of IS/SE, ICH, major GIB, and all major bleeding compared with warfarin. All NOACs have a lower risk of all‐cause mortality compared to warfarin. AMI indicates acute myocardial infarction; CI, confidence interval; GIB, gastrointestinal bleeding; HR, hazard ratio; ICH, intracranial hemorrhage; IS/SE, ischemic stroke/systemic embolism; NOAC, non–vitamin K antagonist oral anticoagulant.

**Figure 6:**
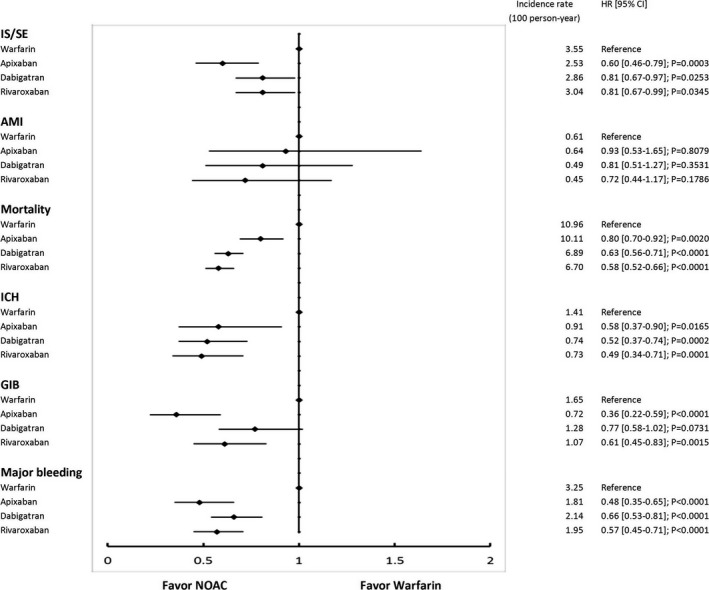
The forest plot of hazard ratios for each low‐dose NOAC (apixaban 2.5 mg twice daily, dabigatran 110 mg twice daily, and rivaroxaban 15/10 mg once daily) vs warfarin comparison. Apixaban, rivaroxaban, and dabigatran are associated with reduced risk of IS/SE, ICH, all major bleeding, and all‐cause mortality compared with warfarin. AMI indicates acute myocardial infarction; CI, confidence interval; GIB, gastrointestinal bleeding; HR, hazard ratio; ICH, intracranial hemorrhage; IS/SE, ischemic stroke/systemic embolism; NOAC, non–vitamin K antagonist oral anticoagulant.

**Figure 7:**
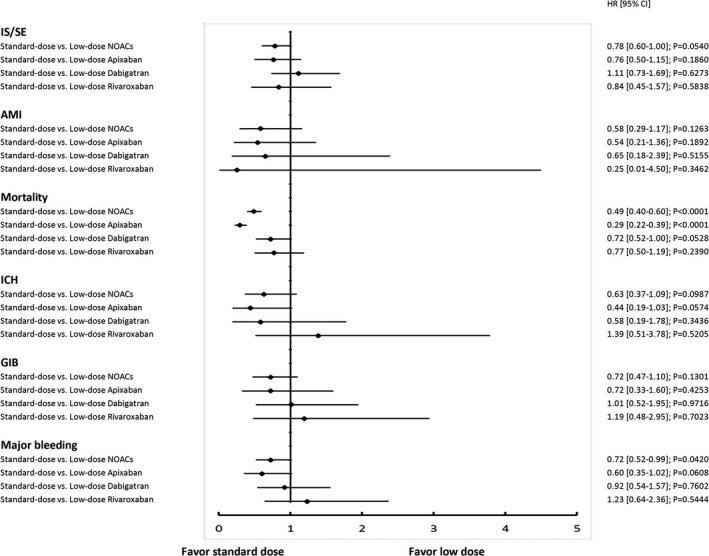
The forest plot of hazard ratios for each standard‐dose NOAC (apixaban 5 mg twice daily, dabigatran 150 mg twice daily, and rivaroxaban 20 mg once daily) vs low‐dose NOAC (apixaban 2.5 mg twice daily, dabigatran 110 mg twice daily, and rivaroxaban 15/10 mg once daily) comparison. In general, standard‐dose NOACs showed 6 comparable outcomes to low‐dose NOACs. It was noted that standard‐dose apixaban had lower risks of all‐cause mortality compared to low‐dose apixaban. AMI indicates acute myocardial infarction; CI, confidence interval; GIB, gastrointestinal bleeding; HR, hazard ratio; ICH, intracranial hemorrhage; IS/SE, ischemic stroke/systemic embolism; NOAC, non–vitamin K antagonist oral anticoagulant.

We further divided each NOAC group into the standard‐dose and low‐dose subgroups. A total of 3623 (62%), 17 760 (88%), and 26 000 (94%) patients were prescribed low‐dose apixaban (2.5 mg twice daily), dabigatran (110 mg twice daily), and rivaroxaban (15 or 10 mg once daily), respectively. Patients who took 3 low‐dose NOACs were older, had higher CHA_2_DS_2_‐VASc and HAS‐BLED scores, and a higher proportion of comorbidities compared with patients who took standard‐dose NOACs (Table 4). Standard‐dose apixaban (5 mg twice daily) was associated with lower risks of IS/SE (HRs [95% CI], 0.45 [0.31–0.65]), ICH (HRs [95% CI], 0.25 [0.12–0.55]), all major bleeding (HRs [95% CI], 0.29 [0.18–0.46]), and mortality (HRs [95% CI], 0.23 [0.17–0.31]) compared with warfarin. The other 2 standard‐dose NOCAs (dabigatran 150 mg twice daily, rivaroxaban 20 mg once daily) showed comparable risks of IS/SE and all major bleeding to warfarin, but lower risk of mortality (HRs [95% CI], 0.45 [0.33–0.63] for dabigatran versus warfarin, and 0.45 [0.30–0.70] for rivaroxaban versus warfarin) (Figure 5). Three low‐dose NOACs showed similar performance as without subgrouping, ie, the risk of IS/SE, ICH, major bleeding and all‐cause mortality were all lower than warfarin (Figure 6). The comparison of standard‐dose NOACs versus low‐dose NOACs were summarized in Figure 7. In general, standard‐dose NOACs showed comparable 6 outcomes to low‐dose NOACs. It was noted that standard‐dose apixaban had a lower risk of all‐cause mortality compared to low‐dose apixaban (HR, 0.29; 95% CI, 0.22–0.39).

**Table 4:** Baseline Characteristics of Patients With NVAF Taking Standard‐Dose or Low‐Dose NOACs

	After Propensity Score Weighting					
	Apixaban		Dabigatran		Rivaroxaban	
	Standard‐Dose 5 mg Twice Daily (n=2220)	Low‐Dose 2.5 mg Twice Daily (n=3623)	Standard‐Dose 150 mg Twice Daily (n=2319)	Low‐Dose 110 mg Twice Daily (n=17 760)	Standard‐Dose 20 mg Once Daily (n=1777)	Low‐Dose 15/10 mg Once Daily (n=26 000)
Age, y	71±9	79±9	71±5	77±5	72±4	76±5
Female	38%	49%	36%	46%	38%	46%
CHA_2_DS_2_‐VASc	3.36±1.49	4.22±1.51	3.48±0.78	3.93±0.82	3.48±0.67	3.91±0.72
HAS‐BLED	2.70±1.11	3.12±1.10	2.83±0.57	2.97±0.59	2.74±0.48	2.97±0.51
Chronic lung disease	9%	16%	11%	13%	11%	14%
Chronic liver disease	16%	16%	18%	16%	16%	16%
Chronic kidney disease	20%	34%	25%	29%	21%	29%
Congestive heart failure	9%	15%	10%	12%	13%	13%
Hypertension	84%	87%	86%	87%	82%	87%
Hyperlipidemia	58%	52%	57%	54%	53%	54%
Diabetes mellitus	40%	42%	41%	41%	41%	41%
Previous stroke	18%	22%	22%	20%	19%	20%
Previous TIA	2%	3%	4%	3%	2%	3%
Ischemic heart disease	12%	14%	12%	13%	12%	13%
Gout	24%	25%	24%	25%	23%	25%
Peripheral artery disease	0%	0%	0%	0%	0%	0%
Malignancy	8%	10%	9%	9%	8%	9%
History of bleeding	1%	3%	1%	2%	2%	2%
Use of NSAIDs	28%	26%	26%	27%	23%	27%
Use of PPI	8%	13%	9%	11%	10%	11%
Use of ACEI/ARB	6%	5%	6%	6%	9%	6%
Use of H_2_ blocker	26%	34%	31%	31%	26%	31%
Use of amiodarone	27%	29%	29%	28%	23%	28%
Use of dronedarone	5%	5%	4%	5%	4%	5%
Use of β‐blocker	60%	58%	62%	58%	60%	59%
Use of diltiazem/verapamil	23%	26%	19%	25%	29%	24%
Use of digoxin	18%	21%	20%	20%	22%	20%
Use of statin	5%	3%	5%	4%	8%	4%
PCI	6%	8%	7%	7%	6%	6%
CABG	0%	1%	1%	0%	0%	0%

ACEI indicates angiotensin‐converting‐enzyme inhibitor; ARB, angiotensin II receptor antagonists; CABG, coronary artery bypass graft; CHA_2_DS_2_‐VASc, congestive heart failure, hypertension, age 75 years or older, diabetes mellitus, previous stroke/transient ischemic attack, vascular disease, age 65 to 74 years, female; HAS‐BLED, hypertension, abnormal renal or liver function, stroke, bleeding history, labile INR, age 65 years or older, and antiplatelet drug or alcohol use (labile INR could not be determined from claims and was excluded from our scoring); NOACs, non–vitamin K antagonist oral anticoagulants; NSAIDs, nonsteroidal anti‐inflammatory drugs; NVAF; nonvalvular atrial fibrillation; PCI, percutaneous coronary intervention; PPI, proton pump inhibitor; and TIA, transient ischemic attack.

## Discussion

This is the largest population‐based study to investigate the efficacy and safety of apixaban, dabigatran, and rivaroxaban versus warfarin with a specific focus on Asians with NVAF during an extended follow‐up period. To date, no previous study has directly compared the effects of these 3 NOACs in Asian patients. Our results showed that all 3 NOACs exhibited lower risks of IS/SE, ICH, all major bleeding, and all‐cause mortality compared with warfarin in a large Asian cohort with AF. We also observed a high prevalence of low‐dose NOACs prescription among the large Asian cohort, with an ≈62%, 88%, and 94% of patients taking low‐dose apixaban, rivaroxaban, and dabigatran, respectively. In contrast to the other 2 standard‐dose NOACs, standard‐dose apixaban (5.0 mg twice daily) demonstrated a lower risk of IS/SE, all major bleeding, and ICH compared with warfarin. All 3 standard‐dose NOACs, apixaban (5.0 mg twice daily), dabigatran (150 mg twice daily), and rivaroxaban (20 mg daily) showed lower mortality than the warfarin group. Three low‐dose NOACs showed similar performance as without subgrouping; that is, the risk of IS/SE, ICH, major bleeding, and mortality were all lower than warfarin.

Few studies have directly compared the efficacy and safety of all 3 NOACs versus warfarin in non‐Asian patients. Larsen et al^22^ compared 3 standard‐dose NOACs versus warfarin in anticoagulant naïve patients with AF using Danish nationwide databases. They concluded that all 3 NOACs and warfarin had a similar risk of ischemic stroke. However, the risk of death or all major bleeding was significantly higher for warfarin and rivaroxaban versus apixaban and dabigatran.^22^ Yao et al^23^ evaluated the efficacy and safety of 3 NOACs by comparing each agent with warfarin using a large US insurance database. Their results indicated that apixaban had lower risks of both stroke and major bleeding and dabigatran had a similar risk of stroke but a lower risk of major bleeding, while rivaroxaban had similar risks of both stroke and major bleeding compared with warfarin.^23^ Graham et al^24^ conducted a retrospective new‐user cohort study with enrollment of 118 891 patients with NVAF using the US Medicare system.^24^ Their data showed that standard‐dose rivaroxaban was associated with a greater number of incidents of ICH and major GIB than standard‐dose dabigatran. In general, most real‐world evidence indicated that dabigatran and apixaban had similar safety profiles, and both were associated with a lower risk of major bleeding than rivaroxaban.

Of note, these real‐world and pivotal studies all focused primarily on non‐Asian patient groups. Previous studies indicated that Asians with AF are more sensitive to warfarin and have an unacceptably higher risk of ICH than non‐Asians even when international normalized ratios are all ideally maintained between 2 and 3.^6^, ^25^, ^26^ Therefore, warfarin has been underdosed or underused in Asians with AF. As expected, Asian patients pay the price with a higher risk of thromboembolic events because of underdosed/underused warfarin as compared with non‐Asians.^9^ Although an international normalized ratios below the therapeutic range of 2 clearly results in a reduced antithrombotic effect, it may be safer in Asians by reducing the warfarin‐related bleeding risk. Unfortunately, Asians still suffer from a higher risk of major bleeding than non‐Asians even when underdosed,^9^ as previous data have shown that the time below therapeutic range can also predict a higher bleeding risk in patients taking warfarin, possibly reflecting a larger variability in international normalized ratio control.^27^

A subgroup analysis of pivotal trials in Asians showed that NOACs displayed better efficacy and safety in Asians than in non‐Asians.^8^ The Asian subgroup analysis from the ARISTOTLE (Apixaban for Reduction in Stroke and Other Thromboembolic Events in Atrial Fibrillation) trial indicated a lower trend of thromboembolic events with apixaban (HR, 0.73; 95% CI, 0.49–1.09) and a significantly lower risk of major bleeding (HR, 0.52; 95% CI, 0.34–0.80) as compared with warfarin.^28^ Our study showed similar results, that is, a significantly lower risk of IS/SE (HR, 0.55; 95% CI, 0.43–0.69) and all major bleeding (HR, 0.41; 95% CI, 0.31–0.53) as compared with warfarin. Dabigatran with 110 mg twice daily was the only low‐dose NOAC that was independently compared with warfarin among the 3 pivotal NOAC studies.^2^ The Asian subgroup analysis from the RE‐LY trial (Randomized Evaluation of Long‐Term Anticoagulation Therapy) indicated that low‐dose dabigatran caused a similar risk of thromboembolic events (HR, 0.82; 95% CI, 0.55–1.24) and a significantly lower risk of major bleeding (HR, 0.57; 95% CI, 0.38–0.86) compared with warfarin.^29^ Our findings in ≈88% of patients taking low‐dose dabigatran in Taiwan are in line with the findings from the RE‐LY trial, with lower risks of thromboembolic events (HR, 0.82; 95% CI, 0.68–0.98) and major bleeding (HR, 0.65; 95% CI, 0.53–0.80) for dabigatran versus warfarin. The subgroup analyses of Asians from the ROCKET‐AF trial (Rivaroxaban Once Daily Oral Direct Factor Xa Inhibition Compared With Vitamin K Antagonism for Prevention of Stroke and Embolism Trial in Atrial Fibrillation) showed a trend toward lower rates of all major bleeding (HR, 0.63, 95% CI, 0.37–1.09) and a similar risk of thromboembolic events (HR, 0.76, 95% CI, 0.42–1.37) for rivaroxaban versus warfarin.^30^ Our data support these trial data with a significantly lower risk of major bleeding (HR, 0.58, 95% CI, 0.46–0.72) and IS/SE (HR, 0.81, 95% CI, 0.67–0.97) for rivaroxaban versus warfarin (Table 5).

**Table 5:** The Summary of the Efficacy and Safety Outcome for the Pivotal Trials and Our Present Study

	ARISTOTLE East Asia Apixaban vs Warfarin			RE‐LY Asia Dabigatran vs Warfarin			ROCKET‐AF East Asia Rivaroxaban vs Warfarin		
	Apixaban	Warfarin	HR (95% CI)	Dabigatran^a^	Warfarin	HR (95% CI)	Rivaroxaban	Warfarin	HR (95% CI)
	Incidence (%/y)	Incidence (%/y)		Incidence (%/y)	Incidence (%/y)		Incidence (%/y)	Incidence (%/y)	
Stroke/systemic embolism	2.52	3.39	0.73 (0.49–1.09)	2.50	3.06	0.82 (0.52–1.24)	2.63	3.38	0.76 (0.42–1.37)
All major bleeding	2.02	3.84	0.52 (0.34–0.80)	2.22	3.82	0.71 (0.56–0.90)	3.44	5.14	0.63 (0.37–1.09)

	Taiwan NHIRD Cohort Apixaban vs Warfarin			Taiwan NHIRD Cohort Dabigatran vs Warfarin			Taiwan NHIRD Cohort Rivaroxaban vs Warfarin		
	Apixaban	Warfarin	HR (95% CI)	Dabigatran	Warfarin	HR (95% CI)	Rivaroxaban	Warfarin	HR (95% CI)
	Incidence (%/y)	Incidence (%/y)		Incidence (%/y)	Incidence (%/y)		Incidence (%/y)	Incidence (%/y)	
Ischemic stroke/systemic embolism	2.26	3.55	0.55 (0.43–0.69)	2.90	3.55	0.82 (0.68–0.98)	3.00	3.55	0.81 (0.67–0.97)
All major bleeding	1.52	3.25	0.41 (0.31–0.53)	2.12	3.25	0.65 (0.53–0.80)	1.97	3.25	0.58 (0.46–0.72)

CI indicates confidential interval; HR, hazard ratio; and NHIRD, National Health Insurance Research Database.

a Dabigatran 110 mg twice daily.

Recent real‐world practice showed a worldwide trend toward the use of low‐dose NOACs.^31^, ^32^, ^33^ Because anticoagulation is usually a preventative treatment in AF patients, physicians tend to play it safe by using low‐dose NOACs in order to prevent bleeding. Similarly, we observed a high prevalence of low‐dose NOAC prescriptions in the present Asian cohort. The smaller body size of Asians as compared with non‐Asians, fear of the iatrogenic bleeding events caused by oral anticoagulants, a high prevalence of elderly patients (mean age of ≈75 years in the present study), and multiple underlying comorbidities and chronic kidney diseases in Asian patients render physicians reluctant to prescribe standard‐dose NOACs for their patients.^10^, ^34^ However, the tendency to prescribe low‐dose NOACs may come at the cost of insufficient effectiveness in stroke prevention. The ORBIT‐AF II registry (Outcomes Registry for Better Informed Treatment of Atrial Fibrillation) indicated that underdosing of NOACs was associated with an increased incidence of cardiovascular hospitalization.^31^ Furthermore, recent studies indicated that off‐label underdosing of apixaban without following the dose‐reduction criteria was associated with a nearly 5‐fold increased risk of stroke.^32^ Interestingly, the reduced efficacy associated with low‐dose apixaban was not seen in those patients treated with either dabigatran or rivaroxaban. Our study did not show a trend of higher thromboembolic events in our patients who took 3 low‐dose NOACs compared with warfarin. Of note, the reported risks of IS/SE per year for apixaban, dabigatran, and rivaroxaban were 2.26%, 2.74%, and 2.84%, respectively, in our study, which was comparable to the primary efficacy outcomes of the 3 NOAC trials (ie, 2.52, 2.50, and 2.63%/year, respectively, in the Asian subgroup analyses) (Table 5). Therefore, the comparable efficacy of low‐dose NOACs versus warfarin in our present study cannot be explained solely by relatively inadequate warfarin control. The optimal dose of NOACs to be used specifically in Asians remains uncertain due to their different body size, pharmacokinetics, and underlying comorbidities as compared with non‐Asians. The J‐ROCKET AF trial (Japanese Rivaroxaban Once Daily Oral Direct Factor Xa Inhibition Compared With Vitamin K Antagonism for Prevention of Stroke and Embolism Trial in Atrial Fibrillation) showed that the pharmacokinetic profiles of a 15‐mg dose of rivaroxaban in Japanese patients was similar to the 20‐mg dose in white patients, indicating that low‐dose rivaroxaban with 15/10 mg is adequate for stroke prevention in AF patients.^35^ Chan et al also reported that low‐dose dabigatran was associated with the lowest risk of ischemic stroke and ICH compared with warfarin with a variety of quality controls in elderly Chinese with AF.^36^, ^37^, ^38^ Further studies are warranted to determine the so‐called “optimal” dose of NOACs in Asians with AF.

### Limitations

The present study had several limitations. We have excluded patients with diagnoses indicating valvular AF, venous thromboembolism, or joint replacement therapy within 6 months before the index date in order to establish a cohort of NVAF taking oral anticoagulants. However, it is not clear that this is sufficiently exclusive. In the present study, we adopted the same definition codes indicating ischemic stroke (*ICD‐9M*: 433, 434, or 436; or *ICD‐10*: I63, I64) as well as previous studies comparing the efficacy of NOACs and warfarin in a real‐world practice. Because the NHIRD does not have brain imaging data, we cannot clarify the issue of whether the reduction of ischemic stroke is contributed from embolic stroke or not. The 3 NOACs prescribed had varying rates of renal excretion and, thus, decisions regarding the use of a specific NOAC or its dosage may have been guided by the renal function and body weight of each patient. Because the NHIRD does not contain creatinine clearance levels, and coding that indicates impaired renal function was dependent on physician's choice, our results may have been biased by the chronic kidney disease population. In addition, we observed a high prevalence of low‐dose NOAC prescriptions in our Asian cohort. The lack of both renal function data and body weights makes it difficult to determine if those patients given low‐dose NOACs were correctly prescribed an adjusted dose or were “off‐label” underdosed. However, the lack of such data is a common limitation of most health insurance databases around the world. Miscoding and misclassification of the underlying comorbidities and outcomes registered by each physician's choice of treatment constitutes an additional limitation of the present study. However, only primary discharge diagnoses were adopted in the present study to improve the outcome accuracy. The 3 NOAC groups also had significantly more comorbidities than the warfarin group. Although inverse propensity score weighting with several variables allowed the matching of comorbidities among the 4 groups, residual confounding by unmeasured variables and selective prescribing behavior could not be excluded in the present study. The Taiwan NHIRD does not have laboratory results, so we had no basis upon which to assess the quality of warfarin anticoagulation among patients. It is possible that the favorable efficacy of NOACs on IS/SE and mortality in our present study was at least partly due to low time in therapeutic range with warfarin. However, this would not explain the reduced risk of IS/SE, ICH, major GIB, and all major bleeding with NOACs at the same time. More importantly, whether quality of warfarin anticoagulation in our study was or was not adequate, it reflects the real‐world practice of anticoagulation quality among Asians taking warfarin. Also, the crude annual risks of IS and major bleeding in the warfarin group in the present study were 2.79% and 3.00%/year, respectively, which were comparable with the Asian post‐hoc analysis of the reported risks with 1.90 and 3.84%/year in the ARISTOLE trial (mean time in therapeutic range of 60.0%), 2.24% and 5.14%/year in the ROCKET‐AF trial (mean time in therapeutic range of 47.1%), and 2.02% and 3.82%/year in the RE‐LY trial (mean time in therapeutic range of 56.5%).^28^, ^29^, ^30^ Apixaban had the shortest mean following‐up period of only 0.76 years due to its introduction later to the market in Taiwan. Although the follow‐up period of apixaban was limited in our present study, our results were compatible with the latest result of Cha et al, showing that apixaban showed a trend of lower risk of IS (HR, 0.67; 95% CI, 0.35–1.17), a significantly lower risk of intracranial hemorrhage (HR, 0.30; 95% CI, 0.09–0.70) and all‐cause mortality (HR, 0.32; 95% CI, 0.18–0.53) compared to warfarin in Koreans with NVAF.^11^ Nevertheless, enrollment of more patients and longer follow‐up periods are necessary to demonstrate the efficacy and safety of Asians taking apixaban in the future. In the present study, the follow‐up period was defined from the index date until the first occurrence of any study outcome or the end date of study period, whichever came first. The same patient could have more than 1 study outcome, and only the study outcome that appeared first was counted because patients with the first study outcome (except for all‐cause mortality) were managed differently afterward. However, it may bias the results if 1 treatment were more likely to cause one of the events because the other events would not be detected. Finally, edoxaban was not included in the study because it was not approved in Taiwan until October 2016.

## Conclusions

Apixaban, rivaroxaban, and dabigatran were associated with reduced risks of IS/SE, ICH, all major bleeding, and all‐cause mortality compared with warfarin in a large Asian cohort with NVAF. There was a high prevalence of low‐dose NOAC prescription among the Asian cohort. In contrast to the other 2 standard‐dose NOACs, standard‐dose apixaban demonstrated a lower risk of IS/SE, all major bleeding, and ICH compared with warfarin. All 3 standard‐dose NOACs showed lower mortality than the warfarin group. Three low‐dose NOACs showed similar performance as without subgrouping.

## Sources of Funding

This study was supported by grants 102‐2628‐B‐182‐011‐MY3, 102‐2314‐B‐182A‐053‐MY3, and 105‐2628‐B‐182A‐003‐MY3 from the Ministry of Science and Technology and CMRPG3B0991‐3, CMRPG3E1681, CMRPG3F0041, CMRPG3F0853, CMRPG3D1631, CMRPD1F0252, CMRPG3F0041 and CMRPG3E0291 from the Chang Gung Memorial Hospital, Linkou, Taiwan.

## Disclosures

None.
